# Effects of Water Immersion on the Internal Power of Cycling

**DOI:** 10.1249/MSS.0000000000002808

**Published:** 2021-10-15

**Authors:** GIOVANNI VINETTI, GUIDO FERRETTI, DAVID HOSTLER

**Affiliations:** 1 Department of Molecular and Translational Medicine, University of Brescia, Brescia, ITALY; 2 Center for Research and Education in Special Environments, Department of Exercise and Nutrition Sciences, University at Buffalo, Buffalo, NY; 3 Department of Anaesthesiology, Clinical Pharmacology, Intensive Care and Emergency Medicine, University of Geneva, Geneva, SWITZERLAND

**Keywords:** AQUATIC EXERCISE, HYDRODYNAMIC RESISTANCE, IMMERSIBLE ERGOMETER, INTERNAL WORK, OXYGEN UPTAKE

## Abstract

**Purpose:**

Water immersion adds additional drag and metabolic demand for limb movement with respect to air, but its effect on the internal metabolic power (*Ė*_int_) of cycling is unknown. We aimed at quantifying the increase in *Ė*_int_ during underwater cycling with respect to dry conditions at different pedaling rates.

**Methods:**

Twelve healthy subjects (four women) pedaled on a waterproof cycle ergometer in an experimental pool that was either empty (DRY) or filled with tap water at 30.8°C ± 0.6°C (WET). Four different pedal cadences (*f*_p_) were studied (40, 50, 60, and 70 rpm) at 25, 50, 75, and 100 W. The metabolic power at steady state was measured via open circuit respirometry, and *Ė*_int_ was calculated as the metabolic power extrapolated for 0 W.

**Results:**

The *Ė*_int_ was significantly higher in WET than in DRY at 50, 60, and 70 rpm (81 ± 31 vs 32 ± 30 W, 167 ± 35 vs 50 ± 29 W, 311 ± 51 vs 81 ± 30 W, respectively, all *P* < 0.0001), but not at 40 rpm (16 ± 5 vs 11 ± 17 W, *P* > 0.99). *Ė*_int_ increased with the third power of *f*_p_ both in WET and DRY (*R*^2^ = 0.49 and 0.91, respectively).

**Conclusions:**

Water drag increased *Ė*_int_, although limbs unloading via the Archimedes’ principle and limbs shape could be potential confounding factors. A simple formula was developed to predict the increase in mechanical power in dry conditions needed to match the rate of energy expenditure during underwater cycling: 44 *f*_p_^3^ – 7 W, where *f*_p_ is expressed in Hertz.

During exercise on a cycle ergometer, net metabolic power (*Ė*) is a linear function of the external mechanical power (W˙) set by the ergometer’s resistance, whose slope is the reciprocal of the delta efficiency (Δη) and whose *y*-intercept is the internal metabolic power (*Ė*_int_) (1–3). From a theoretical standpoint, *Ė*_int_ reflects the fraction of *Ė* used to keep the limbs in motion without generating external forces. Because there is no consensus between biomechanical models on the estimation of internal mechanical power and its interdependence with W˙ (4,5), *Ė*_int_ was suggested as the golden standard reference measurement for the internal power (5). Several studies dissected the determinants of *Ė*_int_, showing that it is a power function of pedaling rate (*f*_p_) (2,6–8), as well as a linear function of limb mass (2) and gravity acceleration (9,10).

Additional energy is also necessary to overcome the resistance to leg movement by the surrounding fluid. This is negligible for air, because of its very small density, and this factor was neglected in all previous models. However, it is not negligible in water, the density of which is 800 times greater than that of ambient air at sea level. The performance of exercise on a cycle ergometer during water immersion shifts the *Ė*–*W˙* relationship upward with respect to air (11–13). According to several authors, *Ė* varies with the third power of *f*_p_ (14–16), as predicted by hydrodynamic analysis of underwater cycling (14,17). However, a comprehensive analysis of *Ė*_int_ during immersed cycling has never been performed, as well as a direct comparison between *Ė*_int_ in water and in air with the same ergometer. Such analysis can allow an advancement in our understanding of the determinants of the increase in *Ė* when cycling in water, as well as in predicting it. In addition to a purely scientific aim, unraveling the effect of *f*_p_, along with other anthropometric factors, could ease and improve the design of future research protocols comparing immersed and dry cycling. In fact, usually water cycling experiments simply assume a fixed 25 W increase in W˙ to account for the increase in *Ė* (13).

Therefore, the aim of this study is to quantify the effect of water resistance on the *Ė*_int_ of cycling at different *f*_p_ in the light- to moderate-intensity domain and to provide predictive equations to estimate the increase in metabolic and mechanical demand.

## METHODS

### Participants

Twelve healthy subjects (four women, eight men) age 28 ± 4 yr (range, 24–36 yr), 73 ± 13 kg heavy (range, 52–94 kg), 173 ± 8 cm tall (range, 163–185 cm), and with a body mass index of 24.2 ± 2.7 kg·m^−2^ (range 19.6–28.2) were recruited from the university community through local advertisement. Ten practiced recreational or competitive sports regularly, whereas two were sedentary. After reviewing the consent document with an investigator, they provided written informed consent. Then, they complete the Physical Activity Readiness Questionnaire for Everyone (2017 PAR-Q+) to rule out contraindications to exercise. Women provided a urine sample for a pregnancy test. A positive result in the questionnaire or in the pregnancy test resulted in exclusion from the study. The study was approved by the University at Buffalo Institutional Review Board (ID: STUDY00003632).

### Instrumental setup

All tests were conducted on an electrically braked cycle ergometer (Pedalmate; Collins, Braintree, MA) adapted to underwater exercise. Continuous air supply from a pressurized tank ensured positive pressure inside the crank case, thus avoiding water infiltration, whereas its electronic control interface was located outside the pool. Subjects were fitted with a two-way non-rebreathing T-shape valve (Hans Rudolph, Shawnee, KS) directing expired air into a mixing chamber via plastic tubing, where oxygen consumption (V˙O_2_) and carbon dioxide production (V˙CO_2_) were calculated employing a heated pneumotachograph, a paramagnetic oxygen analyzer, and an infrared carbon dioxide analyzer (TrueOne 2400; Parvo Medics, Salt Lake City, UT). Subjects also wore an elastic chest strap for HR monitoring (Polar Electro Oy, Kempele, Finland). The ergometer was positioned in an experimental pool that was either empty (DRY condition) or filled with tap water (WET condition) at 30.8°C ± 0.6°C to ensure thermoneutrality for a short period of rest and low- to moderate-intensity exercise (13,18,19). Room air temperature was 23.4°C ± 0.6°C, with a relative humidity of 43% ± 2% and 50% ± 1% in DRY and WET, respectively. In both conditions, subjects wore swim briefs and, to ensure a good and comfortable grip on the pedals, neoprene dive boots, which were tightly sealed at the upper extremity with elastic bands. This clothing configuration was chosen to minimize external interference on the intrinsic drag of the legs. In WET, a 10-kg weight belt was used to counteract buoyancy.

### Protocol

The subjects came into the laboratory on three occasions. In the first visit, weight and height were assessed, and the subject carried out a familiarization trial on the ergometer. The height of the saddle was adjusted and recorded in order to be comfortable and allow water level to be around midsternum. As handlebar, a horizontal tube was attached to the edges of the pool at a custom distance. Then, several short pedaling bouts were performed to experience all the combinations of W˙ and *f*_p_ required by the protocol. Lower limbs volume was assessed with the disc model method (20) from the circumferences measured every 3 cm from the gluteal fold to just above the medial malleolus. The second and third visits consisted of cycling sessions in DRY and WET conditions, the order of which was randomized and balanced. Visits were separated by at least 1 h. Subjects were instructed to be properly hydrated and avoid caffeine and alcohol in the previous 12 h and to eat a light meal 2 to 3 h before arriving at the laboratory. Sessions started with 15 min of rest sitting on the cycle ergometer for instrumentation and recording of the resting metabolic rate. Then, subjects performed four repetitions of incremental exercise, consisting of four consecutive steps of 5 min: 25, 50, 75, and 100 W. Each repetition involved a different *f*_p_: 40, 50, 60, and 70 rpm, the order of which was again randomized and balanced. The rationale of this choice was to ensure that also at the highest intensity (100 W at 70 rpm in WET), the anaerobic component, was negligible, and a metabolic steady state was always attained, so that *Ė* could be estimated by means of gas exchange with minimal error at all workloads. Capillary blood lactate concentration ([La]) was assessed immediately after the last workload and, if higher than 3.5 mM, 15 min after the previous sample (Nova Biomedical, Waltham MA). Repetitions were separated by 15 min of passive recovery or until lactate fell below 2.0 mM. An acoustic metronome was used to help maintain the cadence, which was also checked at the first minute of each workload by recording the time needed to perform 20 to 35 revolutions.

### Data treatment and statistical analyses

Steady-state measurements of HR, V˙O_2_ and V˙CO_2_ were calculated as the average of the last 4 min of rest and the last 2 min of each exercise step. *Ė* was calculated taking into account substrate oxidation with the formula proposed by Jeukendrup and Wallis (21) for low-intensity exercise, converted from kcal·min^−1^ into Watts (309.3 V˙O_2_ + 40.1 V˙CO_2_). Resting energy expenditure was subtracted to the *Ė* of every exercise step to obtain the “net” *Ė*. The individual relationship between net *Ė* and W˙ was treated as linear (net *Ė* = *m* W˙ + *q*), where *q* corresponds to *Ė*_int_ and Δη is calculated as 1/*m* (1). Data are expressed as mean ± standard deviation. Paired sample *t* test was used to compare descriptive physiological variables of the different steps between WET and DRY. Two-way ANOVA for repeated was used to compare the effect of *f*_p_ and water immersion on *Ė*_int_ and Δη, and pairwise comparison with Bonferroni adjustment was performed to locate significant difference. The relationship between *Ė*_int_ and *f*_p_ was treated as a power function with least-squares regression. The level of significance was set at *P* < 0.05. The statistical package Prism (GraphPad Software, La Jolla, CA) was used.

## RESULTS

**FIGURE 1:**
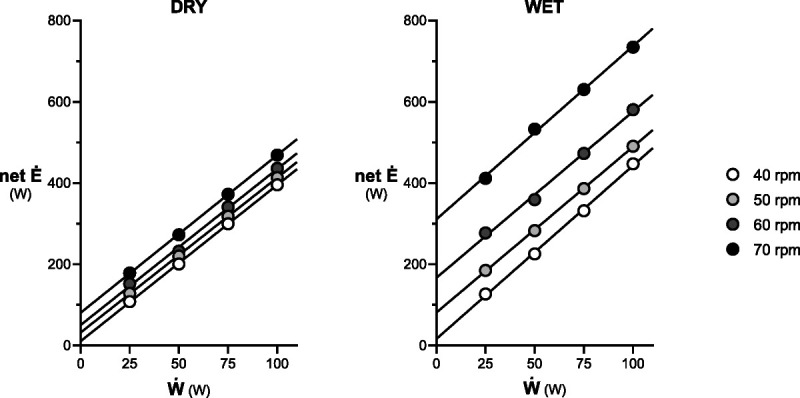
Average net (above resting) metabolic power (net *Ė*) of cycling in air (DRY) and during head-out water immersion (WET) as a function of ergometer’s mechanical power (W˙). The *y*-axis intercept represents the internal metabolic power, whereas the slope is the reciprocal of the Δη.

**FIGURE 2:**
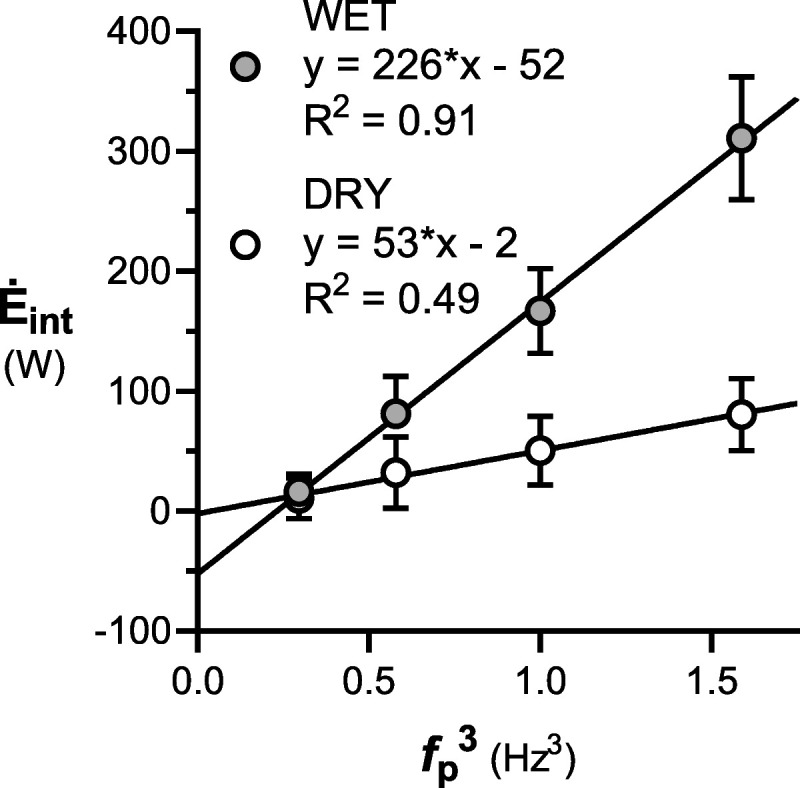
*Ė*_int_ as a *f*_p_^3^ in WET and DRY conditions (*gray* and *white dots*, respectively).

Eight subjects completed the protocol in the same day, the remaining four within 2 d. Difference between measured and target *f*_p_ was 0.1 ± 0.5 rpm (range, +2 to −2 rpm). A clear V˙O_2_ steady state was always attained in all steps. Descriptive physiological data are reported in Table 1. Resting V˙O_2_ and *Ė* did not vary between DRY and WET (*P* = 0.26, *r* = 0.91). During exercise, at all *f*_p_ and at all W˙, V˙O_2_ and *Ė* were higher in WET than in DRY. Peak [La] was lower than 2 mM in all but a few cases in WET. The net *Ė*–*W˙* relationships (Fig. 1) in WET are shifted upward with respect to those in DRY. There was a significant effect of *f*_p_, water immersion, and their interaction on *Ė*_int_ (all *P* < 0.0001), which ranged from a minimum of 11 ± 17 W in DRY at 40 rpm to a maximum of 311 ± 51 W in WET at 70 rpm (Table 2). Δη was independent of *f*_p_ (*P* = 0.78) and significantly affected by water immersion (*P* < 0.0001) but by a marginal extent, being 0.24 to 0.25 in DRY and 0.26 to 0.27 in WET (Table 2). *Post hoc* analysis revealed that *Ė*_int_ was higher in WET than in DRY at 50 to 70 rpm (all *P* < 0.0001) except for 40 rpm (*P* > 0.99). *Ė*_int_ was linearly related to the cube of *f*_p_ (*f*_p_^3^) both in DRY (*R*^2^ = 0.49) and in WET (*R*^2^ = 0.91) conditions (Fig. 2). There was no significant difference between men and women in lower limbs’ volume, *Ė*_int_ and Δη for any condition. Moreover, *Ė*_int_ was not significantly related to lower limb volume, body mass, or body mass index.

**TABLE 1:** Descriptive physiological data of exercise steps of all tests.

			V˙O_2_ (L·min^−1^)	V˙CO_2_ (L·min^−1^)	RER	*Ė* (W)	HR (bpm)	[La] (mM)
	Rest	DRY	0.264 ± 0.050	0.216 ± 0.039	0.82 ± 0.05	90 ± 17	74 ± 12	0.9 ± 0.3
		WET	0.271 ± 0.048	0.224 ± 0.033	0.83 ± 0.04	93 ± 16	65 ± 7*	1.0 ± 0.3
40 rpm	25 W	DRY	0.582 ± 0.083	0.457 ± 0.071	0.78 ± 0.05	198 ± 28	85 ± 12	—
		WET	0.646 ± 0.065*	0.500 ± 0.058*	0.77 ± 0.04	220 ± 22*	89 ± 8	—
	50 W	DRY	0.856 ± 0.087	0.715 ± 0.090	0.83 ± 0.05	291 ± 30	97 ± 14	—
		WET	0.936 ± 0.092*	0.788 ± 0.089*	0.84 ± 0.04	319 ± 32*	101 ± 9	—
	75 W	DRY	1.130 ± 0.097	0.983 ± 0.105	0.87 ± 0.05	390 ± 34	110 ± 16	—
		WET	1.229 ± 0.103*	1.082 ± 0.106*	0.88 ± 0.04	425 ± 36*	113 ± 12	—
	100 W	DRY	1.404 ± 0.112	1.243 ± 0.132	0.88 ± 0.105	486 ± 39	124 ± 18	1.1 ± 0.3
		WET	1.560 ± 0.102*	1.362 ± 0.143*	0.87 ± 0.06	540 ± 37*	127 ± 16	1.3 ± 0.9
50 rpm	25 W	DRY	0.641 ± 0.091	0.509 ± 0.078	0.79 ± 0.04	219 ± 31	89 ± 14	—
		WET	0.815 ± 0.080*	0.644 ± 0.087*	0.79 ± 0.05	278 ± 28*	95 ± 11*	—
	50 W	DRY	0.914 ± 0.091	0.780 ± 0.082	0.85 ± 0.05	311 ± 31	100 ± 15	—
		WET	1.101 ± 0.089*	0.956 ± 0.116*	0.87 ± 0.06	376 ± 32*	106 ± 14*	—
	75 W	DRY	1.187 ± 0.107	1.040 ± 0.105	0.88 ± 0.04	409 ± 37	114 ± 18	—
		WET	1.389 ± 0.112*	1.243 ± 0.133*	0.89 ± 0.05	479 ± 39*	118 ± 16*	—
	100 W	DRY	1.459 ± 0.135	1.313 ± 0.127	0.90 ± 0.05	504 ± 46	126 ± 21	1.2 ± 0.2
		WET	1.686 ± 0.159*	1.543 ± 0.194*	0.91 ± 0.05	583 ± 56*	130 ± 20*	1.4 ± 0.8
60 rpm	25 W	DRY	0.711 ± 0.095	0.570 ± 0.084	0.80 ± 0.04	243 ± 33	93 ± 13	—
		WET	1.080 ± 0.114*	0.903 ± 0.096*	0.84 ± 0.04*	370 ± 39*	107 ± 13*	—
	50 W	DRY	0.982 ± 0.107	0.846 ± 0.100	0.86 ± 0.04	323 ± 36	105 ± 14	—
		WET	1.353 ± 0.128*	1.213 ± 0.133*	0.90 ± 0.05*	452 ± 44*	118 ± 14*	—
	75 W	DRY	1.252 ± 0.136	1.108 ± 0.121	0.89 ± 0.03	432 ± 47	117 ± 15	—
		WET	1.635 ± 0.139*	1.501 ± 0.151*	0.92 ± 0.05*	566 ± 48*	129 ± 17*	—
	100 W	DRY	1.523 ± 0.173	1.382 ± 0.157	0.91 ± 0.04	527 ± 59	130 ± 18	1.1 ± 0.2
		WET	1.940 ± 0.139*	1.832 ± 0.174*	0.94 ± 0.06*	674 ± 48*	143 ± 18*	1.9 ± 1.2*
70 rpm	25 W	DRY	0.787 ± 0.093	0.640 ± 0.092	0.81 ± 0.04	269 ± 32	96 ± 15	—
		WET	1.461 ± 0.167*	1.312 ± 0.184*	0.90 ± 0.06*	505 ± 58	125 ± 16*	—
	50 W	DRY	1.054 ± 0.090	0.922 ± 0.092	0.85 ± 0.04	363 ± 31	108 ± 16	—
		WET	1.805 ± 0.191*	1.682 ± 0.218*	0.91 ± 0.05*	626 ± 67*	137 ± 18*	—
	75 W	DRY	1.340 ± 0.098	1.213 ± 0.106	0.91 ± 0.05	463 ± 34	121 ± 18	—
		WET	2.080 ± 0.178*	1.994 ± 0.232*	0.96 ± 0.06*	723 ± 64*	148 ± 20*	—
	100 W	DRY	1.616 ± 0.115	1.482 ± 0.131	0.92 ± 0.04	559 ± 40	134 ± 21	1.3 ± 0.4
		WET	2.376 ± 0.205*	2.312 ± 0.260*	0.97 ± 0.06*	827 ± 73*	160 ± 22*	3.6 ± 1.8*

*P < 0.05 vs DRY.

**TABLE 2:** Average *Ė*_int_ and Δη in the various conditions.

		40 rpm	50 rpm	60 rpm	70 rpm
*Ė*_int_ (W)	DRY	11 ± 17*	32 ± 30**	50 ± 29**	81 ± 30*
	WET	16 ± 5*	81 ± 31*’***	167 ± 35*’***	311 ± 51*’***
Δη	DRY	0.26 ± 0.02	0.27 ± 0.05	0.27 ± 0.05	0.26 ± 0.03
	WET	0.24 ± 0.02***	0.25 ± 0.05***	0.25 ± 0.03***	0.24 ± 0.03***

*P < 0.05 vs all pedal cadences.

**P < 0.05 vs all pedal cadences except for 50 and 60 rpm.

***P < 0.05 vs DRY.

## DISCUSSION

As predicted, *f*_p_ increased *Ė* in each W˙, with no effect on Δη. The small decrease in Δη in WET is easily explained by the energy dissipated in small rhythmic vertical displacements of the body center of mass at the highest workloads because of the buoyancy and the increased work of immersed breathing (22). In contrast, *Ė*_int_ significantly increased with both *f*_p_ and water immersion. Therefore, the effect of fluid density on the total rate of energy expenditure during cycling was almost exclusively mediated by changes in *Ė*_int_. Before delving into discussion, several limitations should be considered when interpreting our data. First, the underrepresentation of the female sex and of “extremes” in anthropometric characteristics could explain the lack of statistical significance in some of our results. Second, because we relied on the assumption of a pure aerobic metabolism to calculate *Ė*, we could not investigate *f*_p_ greater than 70 rpm in the water. This, together with the provision of longer recovery intervals in case of elevated postexercise [La], was also crucial to minimize fatigue and the slow component of the V˙O_2_ kinetics (23,24) throughout the protocol. However, we still cannot rule out a minimal effect of fatigue, especially in those subjects who completed the study on a single day, although the randomization and balancing of the exercise bouts intrinsically mitigates this possible bias.

Several authors proposed a model of *Ė*_int_ whereby, independent of the type of locomotion, the variation in mechanical energy of limbs with respect to the body center of mass is proportional to the third power of their velocity (6–8,25), that is, the kinetic component of *Ė*_int_ (*Ė*_k_) is given by:


$${\overset{Ė}{}}_{k}=k_{k}\;{f_{p}}^{3}$$ [1]

where *k*_k_ is a proportionality constant, including lower limb mass, the square of a distance that approximately corresponds to the pedal diameter, and muscular efficiency. Our data in air are perfectly described by equation 1 (Fig. 2, DRY). In the case of stationary underwater cycling, additional force is needed to accelerate the surrounding mass of water (the inertial drag, D) in analogy with the “wasted external work” in swimming (25,26), whereas viscous drag forces are negligible (14). Because, in the current model, *Ė*_int_ is assumed as the *y*-intercept of the *Ė*–*W˙* relationship, D is necessarily considered among “internal” forces, at variance with swimming, where it is computed among “external” ones. From a physical standpoint, D is proportional to the square of speed, and the related power is proportional to its cube (14). This supports the concept that also the drag component of *Ė*_int_ (*Ė*_D_) should increase with the cube of *f*_p_:


$${\overset{Ė}{}}_{D}=k_{D}\;{f_{p}}^{3}$$ [2]

where *k*_D_ is a proportionality constant, including the density of the fluid, the lower limb projection area, the drag coefficient, the cube of a distance that approximately corresponds to the pedal diameter, and the muscular efficiency. Equation 2 is in agreement with the previous experimental data (14–16) and current results (Fig. 2, WET). Besides *f*_p_, anthropometric characteristics and sex could not further explain the variance of *Ė*_int_. In DRY, this may be to the small range of pedaling rate investigated (studies in air usually include *f*_p_ ≥ 100 rpm (1,2,6) because they can be sustained in fully aerobic conditions) and the absence of artificial increase in limbs’ mass (2). In WET, this is mainly because of the fact that limb shape, mathematically represented by the drag coefficient, is a major determinant of *k*_D_. However, we were not able to calculate the drag coefficient in the current study.

The negative *y*-intercept of the *Ė*_int_ versus *f*_p_ relationship in WET can be interpreted as a factor accounting for limb unloading due to buoyancy, which explains the absence of differences in *Ė*_int_ between DRY at the lowest *f*_p_ (40 rpm). In fact, investigating the gravitational component of *Ė*_int_ (*Ė*_g_). Bonjour et al. (9) found that:


$${\overset{Ė}{}}_{g}=\varphi \prime \;m_{L}a_{g}\;{f_{p}}^{2}$$ [3]

where *m*_L_ is the mass of the legs, *a*_g_ is the gravity acceleration, and φ’ a proportionality constant. For an average leg density of 1.06 kg·L^−1^ (27), water immersion results in a net acceleration of 0.06 m·s^−2^, so that *Ė*_g_ becomes close to 0 at all *f*_p_ in WET. Although women have typically lower limb density compared with men, the *y*-intercept of the *Ė*_int_ versus *f*_p_ relationship showed no differences between sexes (*P* = 0.61), and it was unrelated to anthropometric characteristics in general. Alternatively, the negative *y*-intercept can be due to a small bias in *Ė*_int_ estimations, especially at lower cadences, where the reduced Δη could underestimate *Ė*_int_. Nevertheless, it can be considered as an empirical factor proportional to fluid density.

Another internal force opposing to movement is the frictional resistance of anatomical structures, which recently has been characterized as mostly viscous (therefore, proportional to speed) and load-dependent (28). Therefore, internal frictional power (*Ė*_f_) is proportional to the square of speed. *Ė*_f_ has been proposed to be of greater importance over *Ė*_k_ during cycling (28); however, because *Ė*_f_ is proportional to *f*_p_^2^, it cannot replace entirely *Ė*_k_, which is indeed proportional to *f*_p_^3^. Interestingly, the *Ė*_f_ theory is compatible with the relationship with *f*_p_^2^ found in hypergravity by Bonjour et al. (9) (equation 3), suggesting that gravity-induced limb loading acts also on internal frictions, contrary to previous hypothesis (10).

In conclusion, the internal power of cycling can be partitioned into several components (*Ė*_k_, *Ė*_f_, *Ė*_g_, *Ė*_D_); however, their reciprocal interdependences make it impossible to express *Ė*_int_ as a mere sum of these components. In air on Earth, fluid density tends to be 0 (in fact, 0.0012 kg·L^−1^ at sea level and 20°C), therefore, *k*_D_ tends to be 0, and *Ė*_D_ can be neglected. In microgravity, also, *a*_g_ is practically 0, therefore, *Ė*_g_ becomes nil and probably also *Ė*_f_ is reduced by some extent. In fact, according to Girardis et al. (10), their subject, who was able to keep the same *f*_p_ at all workloads, had *Ė*_int_ of 28 W in microgravity, which can represent the “unloaded” *Ė*_f_. In water immersion *a*_g_ is close to 0 (in fact, 0.06 m·s^−2^), whereas fluid density is 1.0 kg·L^−1^, therefore, a similar disappearance of *Ė*_g_ and a reduction in *Ė*_f_ are expected, whereas *Ė*_D_ becomes predominant.

### Practical applications

**FIGURE 3:**
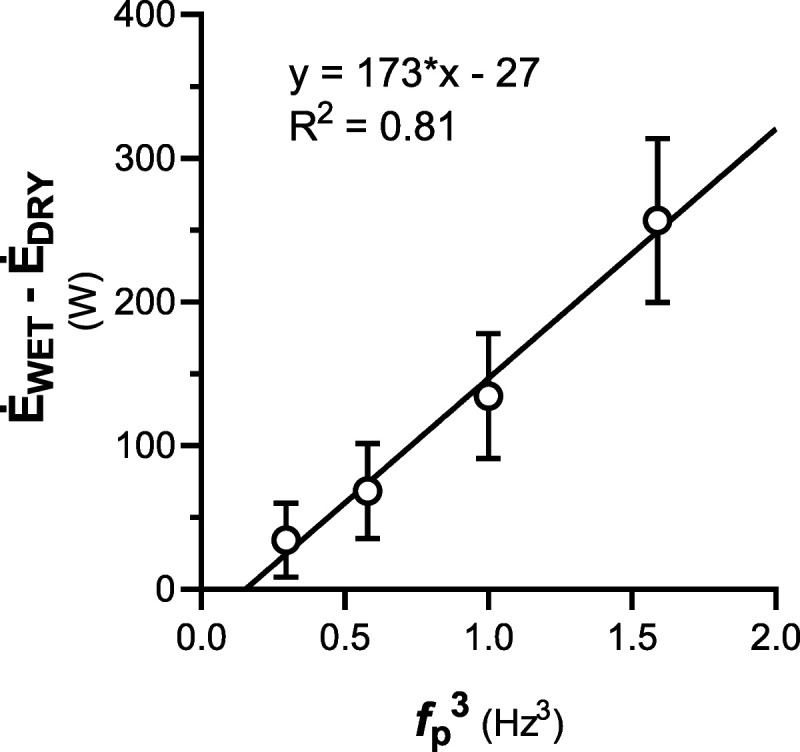
The average difference in metabolic power between of cycling in air and during head-out water immersion, (*Ė*_WET_ − *Ė*_DRY_) as a *f*_p_^3^.


A practical application of this study is evident. The need to quantify the equivalent mechanical power of cycling in water with respect to air could be crucial in several circumstances: the validation and testing of equipment for self-contained underwater breathing apparatus (29,30) and breath-hold diving (31,32), the comparison of the physiological responses between dry and wet exercise, either eupneic (13,33,34) or apneic (35–37), the proper prescription of water-based rehabilitation (33,38,39). In such cases, it could be of help to plot our overall difference in *Ė* between WET and DRY against *f*_p_^3^ (Fig. 3). This difference also cancels out the effect of the type of cycle-ergometer used—provided that it is the same in water as in air. Assuming a Δη of 25.4% (grand average of our Δη values), this could result in a difference in mechanical power of:

$${\overset{W˙}{}}_{\mathrm{WET}}-{\overset{W˙}{}}_{\mathrm{DRY}}=44\;{f_{p}}^{3}-7$$ [4]

where *f*_p_ is expressed in Hertz. Meaning that, for a cadence of 60 rpm (1 Hz), water immersion corresponds to an increase in external load of 37 W. Decreasing to 50 and 40 rpm (0.83 and 0.21 Hz), it becomes 18 and 6 W, respectively. Increasing to 70 or 80 rpm (1.67 and 1.33 Hz), it goes up to 63 and 97 W, respectively. Although *f*_p_ can explain 81% of the variance of the increase in *Ė* (*R*^2^ = 0.81), some interindividual differences still remain unexplained. Although Δη has typically little variability, especially within our range of *f*_p_ and exercise intensities (40), other anatomical or neuromuscular factors could affect the generalizability of our results in populations with impaired locomotor function or extremes anthropometric characteristics, such as the elderly, the disabled, or the morbid obese. Finally, caution should be used in extrapolating our results to cycling far above 70 rpm or far below 40 rpm, as well as while wearing bulkier clothes (i.e., swim trunks, wetsuit). Nevertheless, this study represents a step forward from the traditional finding that water cycling corresponds to a 25 W increase in W˙ (13), which proves true only for a *f*_p_ of 54 rpm and can be better refined by equation 4 taking into account *f*_p_.
